# Racial and Ethnic Disparities at End of Life Among Persons With Alzheimer’s Disease and Related Dementias

**DOI:** 10.1093/geroni/igaa057.885

**Published:** 2020-12-16

**Authors:** Morgan Rising, Nicole Hair

**Affiliations:** 1 University of South Carolina, Columbia, United States; 2 University of South Carolina, Columbia, South Carolina, United States

## Abstract

End-of-life (EOL) care for persons with Alzheimer’s disease and related dementias (ADRD) is heterogeneous and, often, inconsistent with patients’ priorities. While differences in physician-patient communication or the health care decision-making process likely contribute to disparities in EOL care, little is known about the relationships between race, advance care planning (ACP), and quality of care among persons with ADRD. The aim of this study was to (1) characterize trends in both ACP and EOL treatment intensity in persons with ADRD and (2) test whether racial differences in ACP mediate disparities in EOL care. We analyzed a population-based cohort of older adults with cognitive impairment or dementia who participated in the Health and Retirement Study (HRS) and died between 2000 and 2014 (n = 5,316). While participation in ACP among persons with ADRD increased from 2000 to 2014 (66% to 83%, P < 0.05), models stratified by race showed that differences in participation rates across white and nonwhite persons with ADRD persisted over the sample period. Racial disparities in the location of death, a proxy for the intensity of EOL care, narrowed from 2000 to 2014. However, next-of-kin surrogates of nonwhite persons with ADRD were much more likely to report the decedent received “all care possible…in order to prolong life”. Assignment of a durable power of attorney was found to influence location of death, while both creation of a living will and participation in discussions about EOL care preferences were found to influence the likelihood that decedents received all possible life-prolonging treatments.

